# Supplementary material for: Development and In Vitro and In Vivo Evaluation of an Antineoplastic Copper(II) Compound (Casiopeina III-ia) Loaded in Nonionic Vesicles Using Quality by Design
Source: Int J Mol Sci. 2022 Oct 22;23(21):12756. doi: 10.3390/ijms232112756 (PMC9655312; doi:10.3390/ijms232112756)
Supplement: Supplementary file 1 [file ijms-23-12756-s001.zip › ijms-1954334-supplementary.pdf]

International Journal of *Molecular Sciences*

Article

Development and In Vitro and In Vivo Evaluation of an Antineoplastic Copper(II) Compound (Casiopeina III-ia) Loaded in Nonionic Vesicles Using Quality by Design

Zenayda Aguilar-Jiménez, Mauricio González-Ballesteros, Silvia G. Dávila-Manzanilla, Adrián Espinoza-Guillén and Lena Ruiz-Azuara*

Facultad de Química, Departamento de Química Inorgánica y Nuclear, Universidad Nacional Autónoma de México, Av. Universidad 3000, México, DF 04510, México.

* Correspondence: lenar701@gmail.com

Citation: Aguilar-Jiménez, Z.; González-Ballesteros, M.; Dávila-Manzanilla, S.G.; Espinoza-Guillén, A.; Ruiz-Azuara, L. Development and In Vitro and In Vivo Evaluation of an Antineoplastic Copper(II) Compound (Casiopeina III-ia) Loaded in Nonionic Vesicles Using Quality by Design. *Int. J. Mol. Sci.* **2022**, *23*, 12756. https://doi.org/10.3390/ijms232112756

Academic Editor: Michael Danquah

Received: 20 September 2022
Accepted: 19 October 2022
Published: 12 October 2022

Publisher's Note: MDPI stays neutral with regard to jurisdictional claims in published maps and institutional affiliations.

Copyright: © 2022 by the authors. Submitted for possible open access publication under the terms and conditions of the Creative Commons Attribution (CC BY) license (https://creativecommons.org/licenses/by/4.0/).

Table S1. Plackett-Burman design.

level	Evaporation		Injection				Formulation						
	A	B	C	D	E	F	G	H	I	Y_1	Y_2	Y_3	Y_4
-1	30	50	40	10	50	2.5	60	200	2.5				
0	60	100	50	30	100	3.75	130	600	3.75				
1	90	150	60	50	150	5	200	1000	5				
Run	A	B	C	D	E	F	G	H	I				
1	1	-1	1	-1	-1	-1	1	1	1	219 ± 29.80	0.478 ± 0.08	-234	07.53 ± 1.03
2	1	1	-1	1	-1	-1	-1	1	1	229 ± 01.87	0.279 ± 0.00	-333	19.65 ± 0.08
3	-1	1	1	-1	1	-1	-1	-1	1	227 ± 01.90	0.378 ± 0.00	-331	31.07 ± 0.09
4	1	-1	1	1	-1	1	-1	-1	-1	139 ± 02.10	0.214 ± 0.01	-307	21.44 ± 0.10
5	1	1	-1	1	1	-1	1	-1	-1	206 ± 05.00	0.317 ± 0.04	-284	16.33 ± 0.14
6	1	1	1	-1	1	1	-1	1	-1	321 ± 07.86	0.354 ± 0.05	-342	40.94 ± 0.05
7	-1	1	1	1	-1	1	1	-1	1	189 ± 09.01	0.435 ± 0.03	-281	10.98 ± 0.82
8	-1	-1	1	1	1	-1	1	1	-1	199 ± 09.89	0.421 ± 0.06	-269	19.19 ± 0.77
9	-1	-1	-1	1	1	1	-1	1	1	340 ± 03.65	0.281 ± 0.01	-333	19.68 ± 0.35
10	1	-1	-1	-1	1	1	1	-1	1	123 ± 00.38	0.268 ± 0.01	-255	10.79 ± 0.79
11	-1	1	-1	-1	-1	1	1	1	-1	329 ± 08.98	0.403 ± 0.02	-328	15.36 ± 0.07
12	-1	-1	-1	-1	-1	-1	-1	-1	-1	197 ± 02.88	0.303 ± 0.02	-332	22.86 ± 0.13
13	0	0	0	0	0	0	0	0	0	230 ± 05.97	0.367 ± 0.04	-310	16.35 ± 0.26
14	0	0	0	0	0	0	0	0	0	190 ± 02.32	0.240 ± 0.00	-277	10.50 ± 0.17
15	0	0	0	0	0	0	0	0	0	236 ± 02.88	0.365 ± 0.03	-312	16.90 ± 0.08

Evaporation: **A**: time (min); **B**: speed rate (rpm) Injection: **C**: temperature (°C); **D**: injection rate (mL/hora); **E**: speed rate (rpm); **F**: solvent volume (mL) Formulation: **G**: Cas III-ia concentration (μM); **H**: S/C/P concentration (μM); **I:** P concentration. **Particle size:** Y_1 **PDI**: Y_2 **Superficial charge**: Y_3 **Encapsulation efficiency**: Y_4. Where S/C/P is Span60/cholesterol/Pluronic F127.

Table S2. Factorial and Central Composite Design for the optimization of the casiopeina nanoformulation. **Particle size**: Y_1 **PDI:** Y_2 **Superficial charge:** Y_3 **Encapsulation efficiency:** Y_4. Where S/C/P is Span60/cholesterol/Pluronic F127.

Level		S/C/P concentration	Cas III-ia concentration	Speed rate	Y_1	Y_2	Y_3	Y_4
-1		200	60	50				
0		600	130	100				
1		1000	200	150				
Run	Block							
1	1	1	-1	1	144 ± 2.20	0.19 ± 0.023	-332	40.44 ± 0.22
2	1	-1	-1	-1	158 ± 3.84	0.22 ± 0.027	-341	29.52 ± 0.29
3	1	0	0	0	223 ± 2.15	0.25 ± 0.020	-308	17.78 ± 0.97
4	1	-1	1	1	163 ± 1.24	0.22 ± 0.013	-301	11.26 ± 0.25
5	1	1	1	-1	350 ± 2.92	0.27 ± 0.006	-293	04.50 ± 0.33
6	3	1	-1	1	169 ± 0.21	0.21 ± 0.006	-392	35.72 ± 0.27
7	3	-1	-1	-1	166 ± 2.63	0.22 ± 0.028	-404	24.46 ± 0.29

8	3	-1	1	1	143 ± 2.70	0.26 ± 0.025	-315	15.02 ± 0.26
9	3	1	1	-1	387 ± 11.24	0.46 ± 0.047	-293	04.21 ± 0.40
10	3	0	0	0	204 ± 2.62	0.27 ± 0.007	-345	14.96 ± 1.34
11	2	-1	1	-1	178 ± 3.23	0.30 ± 0.039	-292	15.38 ± 0.37
12	2	1	1	1	170 ± 2.31	0.30 ± 0.019	-305	15.36 ± 0.56
13	2	-1	-1	1	148 ± 2.06	0.23 ± 0.009	-379	28.98 ± 0.31
14	2	0	0	0	209 ± 3.58	0.31 ± 0.038	-320	13.40 ± 0.83
15	2	1	-1	-1	140 ± 0.87	0.24 ± 0.006	-356	32.35 ± 0.50
16	4	1	1	1	233 ± 4.26	0.32 ± 0.029	-312	07.61 ± 0.18
17	4	-1	1	-1	233 ± 3.75	0.35 ± 0.051	-275	09.90 ± 0.21
18	4	-1	-1	1	157 ± 1.83	0.33 ± 0.042	-371	30.34 ± 0.08
19	4	0	0	0	240 ± 2.20	0.26 ± 0.003	-303	09.64 ± 0.50
20	4	1	-1	-1	174 ± 4.09	0.31 ± 0.012	-365	30.83 ± 0.11
21	--	40	130	100	189 ± 7.00	0.25 ± 0.008	-342	20.21 ± 0.57
22	--	1160	10	100	223 ± 2.00	0.23 ± 0.010	-297	23.06 ± 0.54
23	--	600	32	100	149 ± 0.66	0.21 ± 0.008	-377	35.56 ± 0.06
24	--	600	228	100	260 ± 2.06	0.27 ± 0.015	-307	12.69 ± 0.24
25	--	600	130	30	298 ± 6.72	0.29 ± 0.010	-358	27.77 ± 0.32
26	--	600	130	170	267 ± 0.50	0.26 ± 0.011	-351	12.06 ± 0.00
27	--	600	130	100	246 ± 3.11	0.36 ± 0.006	-341	22.77 ± 1.23
28	--	600	130	100	247 ± 4.43	0.29 ± 0.043	-360	19.69 ± 1.79

Table S3. Regression equation in uncoded units obtained through composite central design.

Regression equation	R^2
Particle Size = 57.3 + 0.1578*A +1.868*B - 0.581*C - 0.000130*A^2 -0.00441*B^2 + 0.00723*C^2 + 0.0 00952*A*B - 0.00066*A*C -0.00748*B*C	95.13%
PDI = 0.1720 + 0.000082*A + 0.0011*B + 0.00011*C -0.000002*B^2 + 0.00002*C^2 + 0.000001*A*B - 0.000001*A*C - 0.000004*B*C	66.59%
Surface charge= - 455 0.0263*A + 0.931*B + 0.925*C + 0.000043*A^2 -0.00093*B^2 - 0.00437*C^2 + 0.000156*A*B + 0.000062*A*C -0.00129*B*C	76.87%
EE = 46.75-0.0060*A - 0.2448*B -0.101*C + 0.000012*A^2 + 0.000643*B^2 + 0.000402*C^2 - 0.000103*A*B + 0.000065*A*C -0.000054*B*C	88.79%
A: S/C/P concentration, B: Cas III-ia concentration, C: speed rate	

Table S4.- Correlation coefficients and kinetic constants of kinetic models

**Model**	R^2	**k**
Zero-order	0.8327	ko: 1.2065
First order	0.9052	k_1 :0.0861
Higuchi	0.9370	k_H :8.6796
Korsmeyer-Peppas	0.9833	k= 16.15 n=0.16

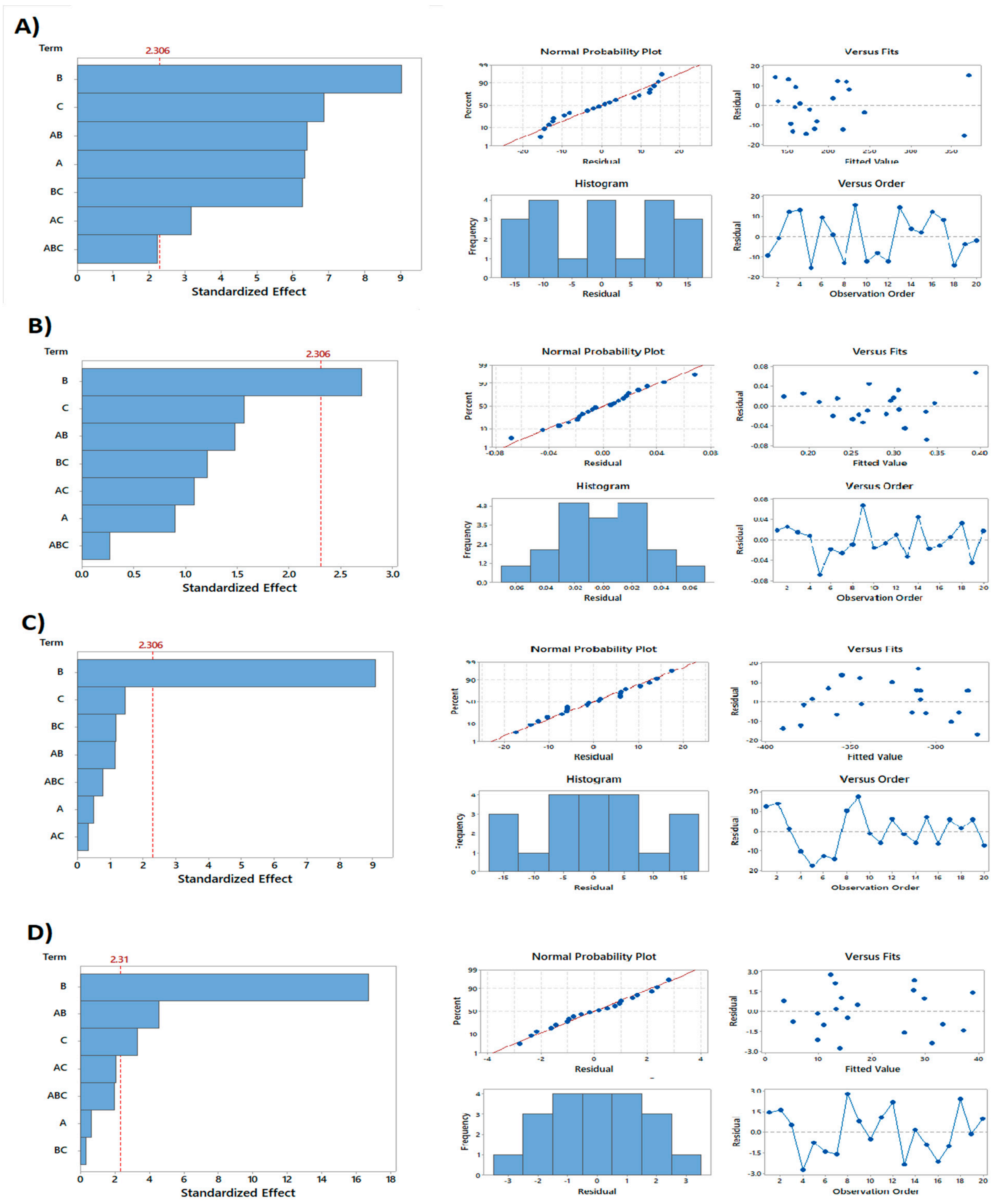

Figure S1. Pareto charts, residual and normality plots of the standardized effects ($\alpha = 0.05$) of factors for factorial design, where A) particle size, B) PDI, C) surface charge, and D) encapsulation efficiency for Factorial design. Where **A**: S/C/P concentration, **B**: Cas III-ia concentration, **C**: Speed rate

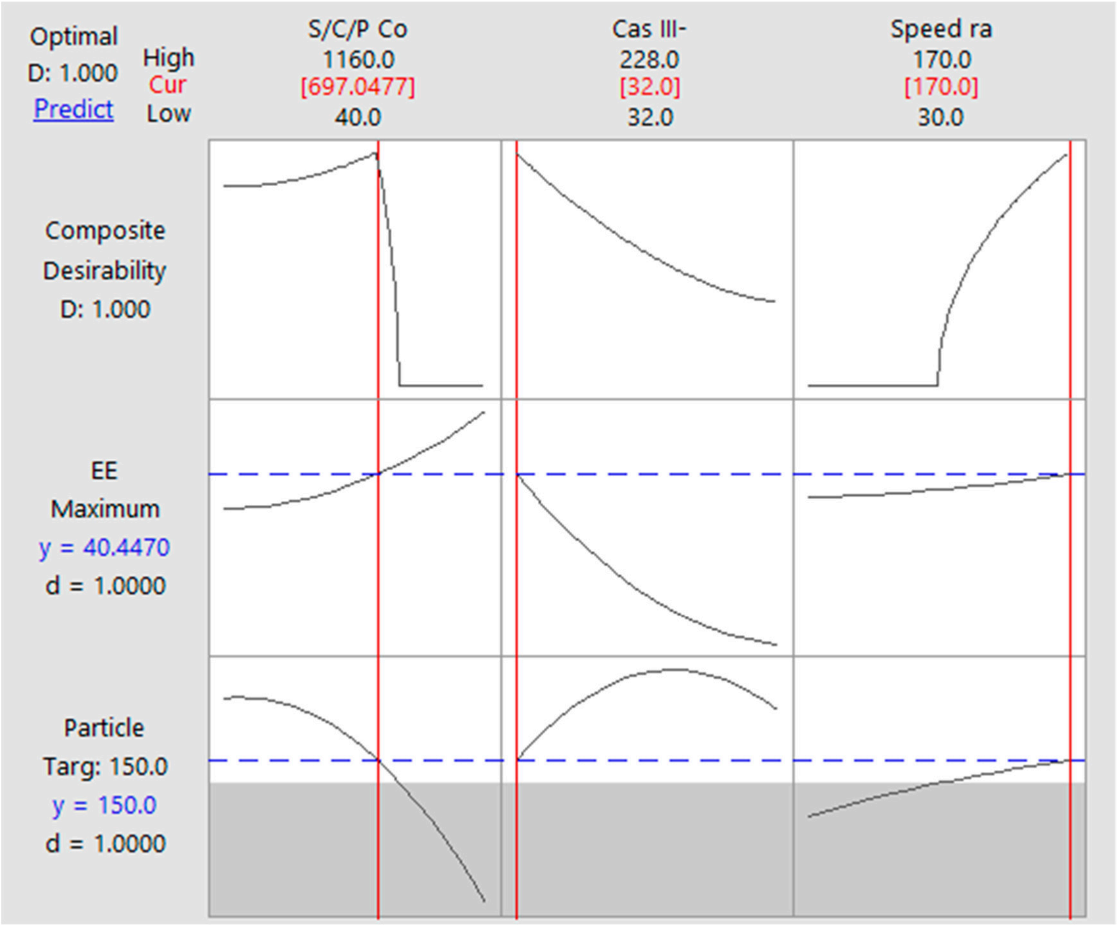

Response	Fit	DE Fit	95 % IC	95 % IP
EE	40.44	3.87	(32.14, 48.73)	(28.35, 52.52)
Particle size	150.0	19.1	(109.0, 191.0)	(90.2, 209.8)

Figure S2. Response optimizer plot from central composite design for niosomes with CasIII-ia.

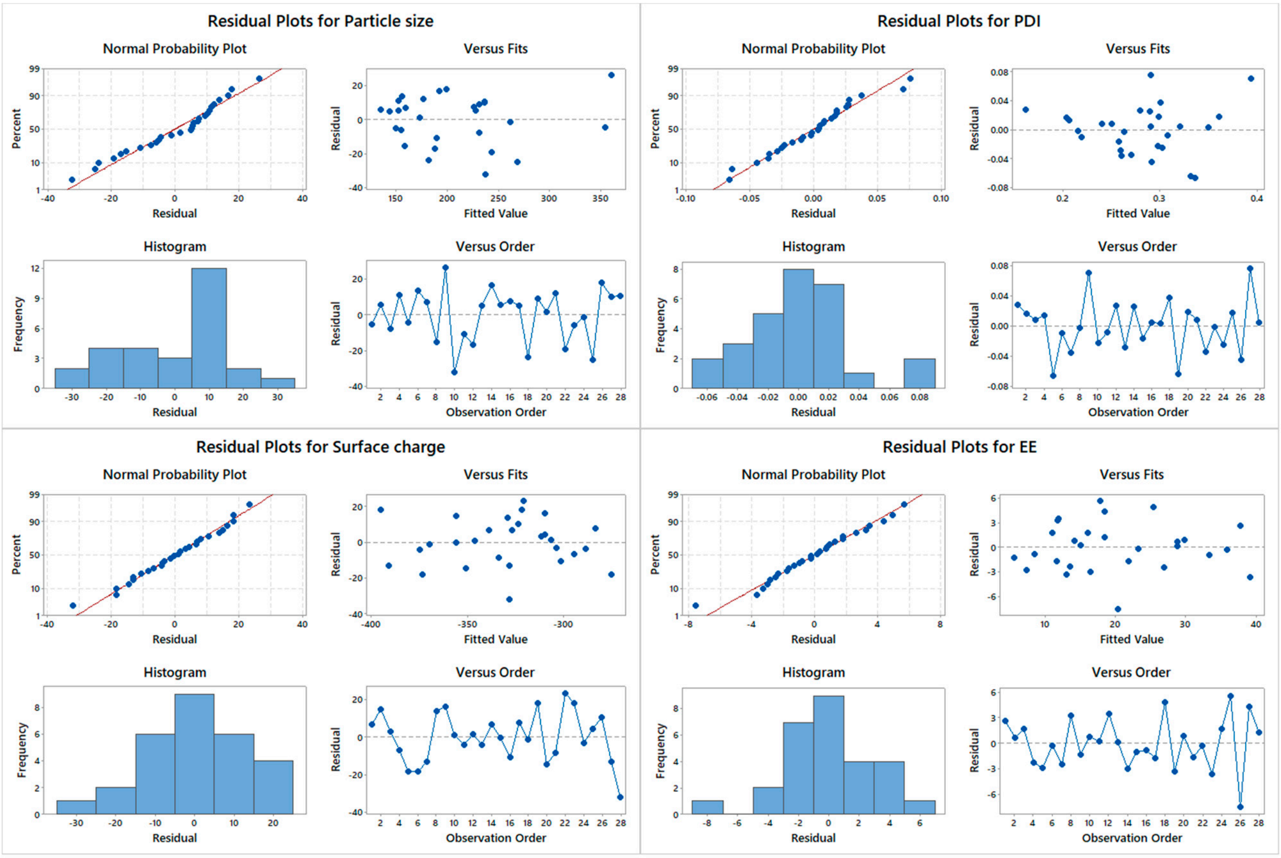

Figure S3. Residual and normality plots of the standardized effects ($\alpha = 0.05$) of factors for the central composite design, where A) particle size, B) PDI, C) surface charge and D) encapsulation efficiency for Factorial design. Where **A**: S/C/P concentration, **B**: Cas III-ia concentration, **C**: Speed rate

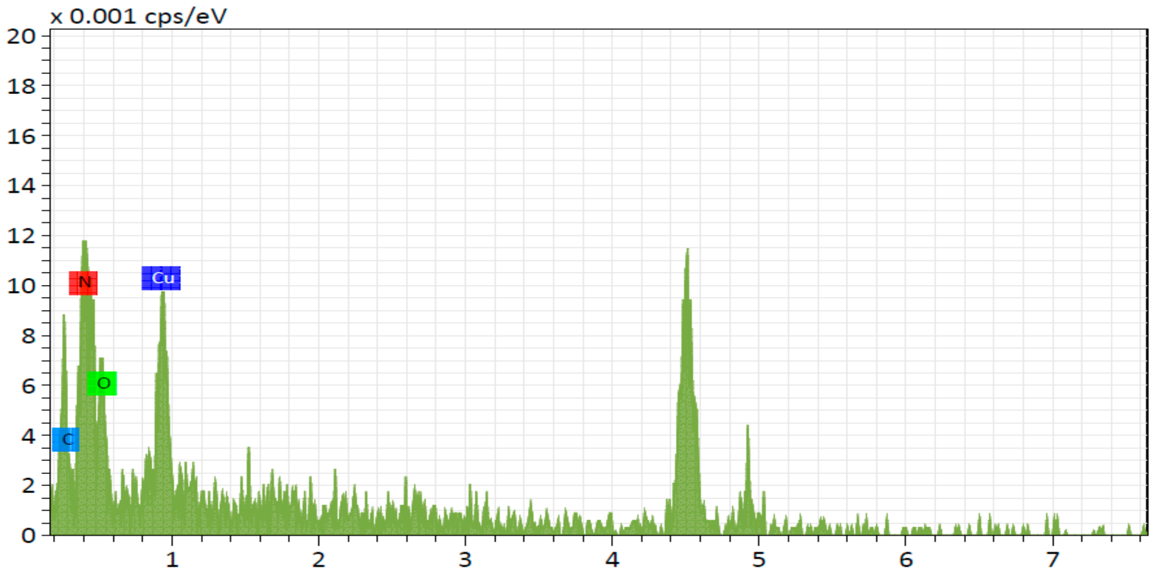

Figure S4. Energy-Dispersive Spectroscopy (EDS) for niosomes with Cas III-ia

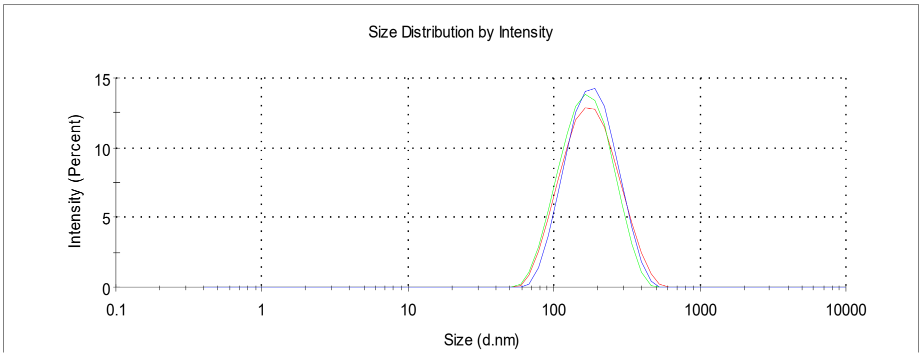

Figure S5. Particle size for niosome with Cas III-ia from dynamic light scattering

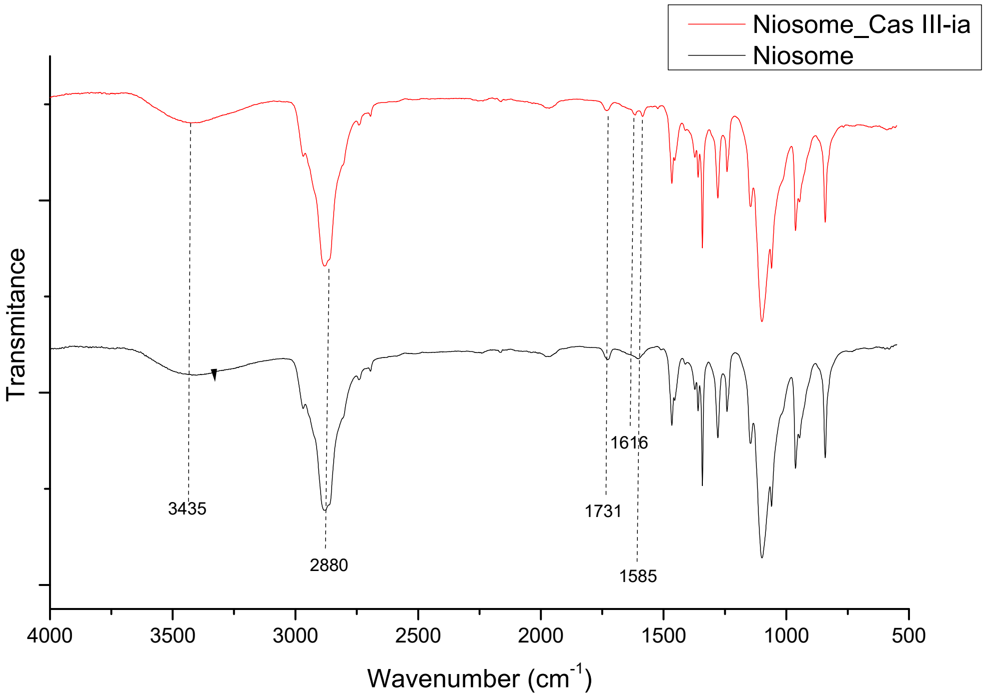

Figure S6. FTIR-ATR spectra of Cas III-ia and niosome with Cas III-ia.
